# Low Doses of Methylmercury Induce the Proliferation of Thyroid Cells In Vitro Through Modulation of ERK Pathway

**DOI:** 10.3390/ijms21051556

**Published:** 2020-02-25

**Authors:** Valentina Maggisano, Stefania Bulotta, Marilena Celano, Jessica Maiuolo, Saverio Massimo Lepore, Luana Abballe, Michelangelo Iannone, Diego Russo

**Affiliations:** 1 Department of Health Sciences, University “Magna Graecia” of Catanzaro, 88100 Catanzaro, Italy; vmaggisano@unicz.it (V.M.); bulotta@unicz.it (S.B.); celano@unicz.it (M.C.); jessicamaiuolo@virgilio.it (J.M.); sm.lepore78@gmail.com (S.M.L.); 2 Department of translational and precision medicine, “Sapienza” University of Rome, 00161 Rome, Italy; luana.abballe@uniroma1.it; 3 National Council of Research (CNR), Institute of Neurological Sciences, 88100 Catanzaro, Italy; michelangelo.iannone@gmail.com

**Keywords:** endocrine disruptors, thyroid cancer, mercury, environmental contaminants

## Abstract

Exposure to environmental endocrine disruptors has been associated with an increased frequency of thyroid pathology. In this study, we evaluated the effects of various concentrations of methylmercury (MeHg) on immortalized, non-tumorigenic thyroid cells (Nthy-ori-3-1). Exposure to MeHg at 2.5 and 5 µM for 24 h caused a reduction in cell viability with a decrease of the cell population in sub-G0 phase, as detected by MTT and flow cytometry. Conversely, MeHg at the lower concentration of 0.1 µM increased the cell viability with a rise of G2/M phase. An immunoblot analysis showed higher expression levels of phospho-ERK and not of phospho-Akt. Further enhancement of the cell growth rate was observed after a prolonged exposure of the cells up to 18 days to MeHg 0.1 µM. The present findings demonstrate the toxicity of high concentrations of MeHg on thyroid cells, while showing that treatment with lower doses of Hg, as may occur after prolonged exposure to this environmental contaminant, exerts a promoting effect on thyroid cell proliferation, by acting on the ERK-mediated pro-oncogenic signal transduction pathway.

## 1. Introduction

In the last few years, thyroid cancer incidence has increased significantly [1]. The presence of tumors of all sizes excludes that this increment is due only to more intensive and sensitive diagnostic procedures and suggests the involvement, as additional causative factors, of environment or lifestyle changes [2]. Heavy metals in the water and in the atmosphere, which characterize the environmental pollution, are known to act as endocrine disruptors (ED), and, by inducing cell oxidative stress and altering the DNA, may promote malignant transformation. In particular, the mercury compounds induce severe toxic effects both in human and animal experimental models [3,4], behaving as ED even against thyroid tissue [5,6]. Moreover, high levels of Hg, recently detected together with other heavy metals in volcanic areas [7,8], have been suspected to play a co-causative role in the high incidence of thyroid cancer, as earlier proposed by Zaichick et al. [9] However, to date, the molecular alterations occurring in thyroid cells exposed to environmental heavy metals, able to promote the transformation and/or the selective growth of neoplastic cells, are not known, as well as the doses and duration of exposure necessary for such an action [10].

In the present study, we analyzed the effects of methylmercury (MeHg) on immortalized, non-tumorigenic ‘normal’ thyroid cells Nthy-ori-3-1 and the molecular changes occurring after exposure to MeHg.

## 2. Results

### 2.1. Effects of Short Treatment of Nthy-ori-3-1 Cells with MeHg on the Growth and Cell Cycle

**Figure 1:**
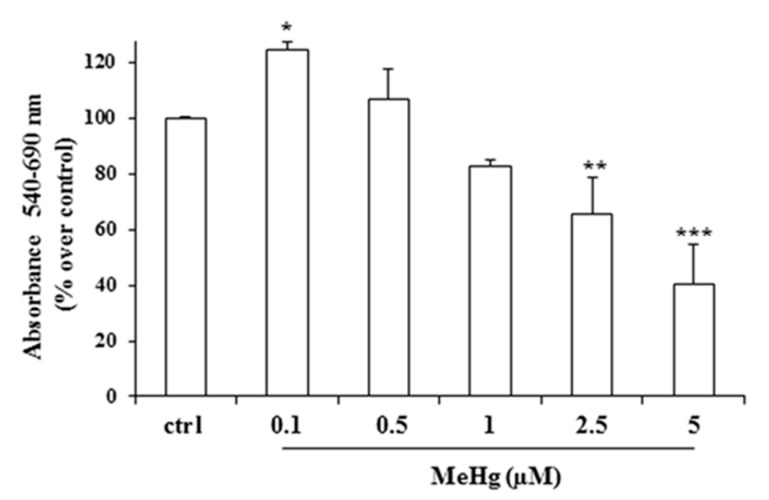
Effects of short treatment with methylmercury (MeHg) on cell viability. Nthy-ori-3-1 cell viability was measured by MTT assay after 24 h of treatment with various concentrations of MeHg. Results are expressed as percentage of increment vs. untreated cells, representing the mean ±SD of three independent experiments. Statistical analysis was performed using the Tukey–Kramer multiple comparisons test. * *p* < 0.05, ** *p* < 0.01, *** *p* < 0.001, vs. untreated cells (control).

**Figure 2:**
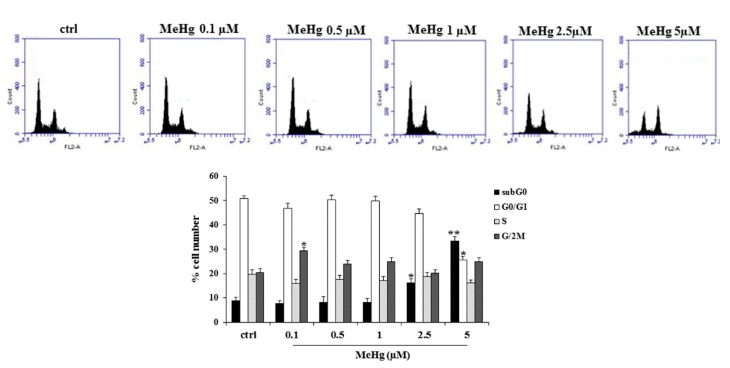
Effects of MeHg on cell cycle progression of Nthy-ori-3-1 cells. Cells were exposed for 24 h to the indicated concentrations of MeHg and analyzed by flow cytometry as described in Methods. Untreated cells were used as control (control). In upper panel, cytofluorimetric graphics of a representative experiment of three separate determinations are shown. Lower panel shows the distribution of cells in subG0, G0/G1, S and G2/M phases of the cellular cycle. The results represent the mean ± SD of three independent experiments. Statistical analysis was performed using the Tukey–Kramer multiple comparisons test. * *p* < 0.05, ** *p* < 0.01, vs. control. No effect was detected on the production of reactive oxygen species (ROS) (Figure S1).

We first analyzed the effects of various concentrations of MeHg on the proliferation of Nthy-ori-3-1 cells. Treatment at the concentrations of 2.5 and 5 µM of MeHg for 24 h caused a significant reduction of cell viability of about 40% and 60% vs. untreated cells, respectively (Figure 1). Conversely, MeHg at the concentration of 0.1 µM determined a significant increase of Nthy-ori-3-1 cell viability (about 20% vs. untreated cells) (Figure 1). Next, we evaluated the effects of MeHg on cell cycle progression. After 24 h of treatment, cytofluorimetric analysis showed the presence of a rise in G2/M phase of the cell cycle at a concentration of 0.1 µM, and an increase of cell population in sub-G0 phase when MeHg was added at higher concentrations (5 µM) (Figure 2).

### 2.2. Effects of Short Treatment with MeHg on ERK and Akt Pathways and Expression of Thyrocyte Differentiation Markers

**Figure 3:**
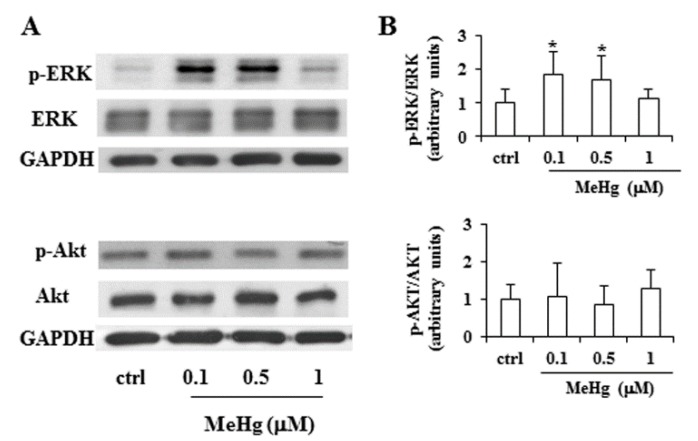
Effects of low doses of MeHg on ERK and Akt phosphorylation. Nthy-ori-3-1 cells were treated for 6 h in the absence or presence of MeHg, at the concentrations of 0.1, 0.5 and 1 µM. (**A**) Immunoblot analysis of active, phosphorylated ERK (p-ERK) and phosphorylated Akt (p-Akt), and total form of the enzymes (ERK, Akt), was performed by western blotting. GAPDH was used as loading control. (**B**) Densitometric analysis from four immunoblot of p-ERK/ERK and p-AKT/AKT. Values are expressed as a ratio over the loading control (arbitrarily assigned as 1). Untreated cells were used as control and indicated as control. Statistical analysis was performed using the Tukey–Kramer multiple comparisons test. * *p* < 0.05 vs. control.

To clarify the molecular mechanism involved in the growth promoting effect of low concentrations of MeHg, we investigated whether short exposure to 0.1 and 0.5 µM MeHg would modulate two major signal transduction pathways involved in the control of thyrocyte proliferation. In Nthy-ori-3-1 cells, treatment with MeHg at 0.1 and 0.5 µM for 6 h determined an increase of ERK phosphorylation, without modifying the total levels of these proteins (Figure 3). In the same experimental conditions, no variation of phospho-Akt levels was observed (Figure 3).

Furthermore, after treatment with MeHg 0.1 µM, the gene expression of the main differentiation markers of thyroid cells was analyzed and no changes in *Thyroglobulin* (*Tg)* and *NIS* levels were observed (Figure S2). No detectable levels of *thyroid-stimulating hormone receptor* (TSHR) or *Thyroperoxidase* (TPO) were observed in Nthy-ori-3-1 cells, treated or not with MeHg (Figure S2).

### 2.3. Effects of Prolonged Treatment with MeHg on the Growth of Nthy-ori-3-1 Cells

**Figure 4:**
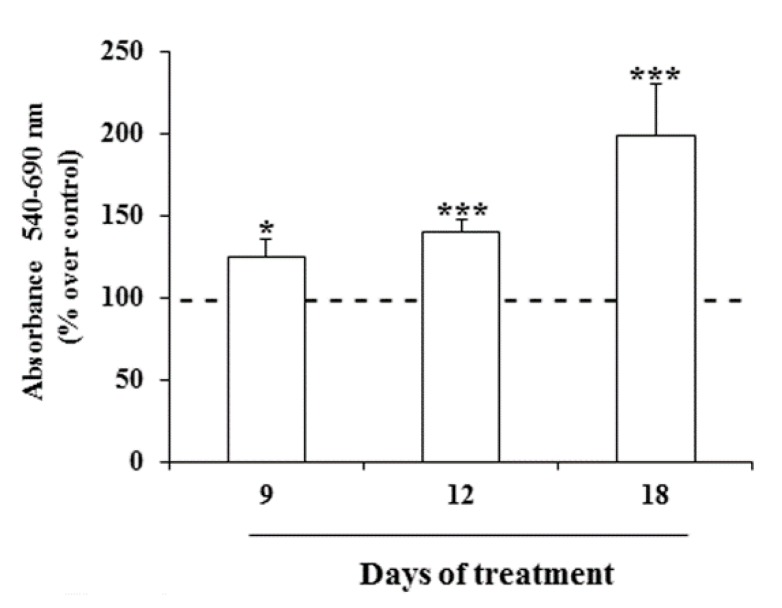
Effects of prolonged treatment with MeHg on cell viability. Nthy-ori-3-1 cell growth was determined by MTT assay after treatment for 9, 12 and 18 days with MeHg 0.1 µM. Results are expressed as percentage of increment vs. untreated cells, considered arbitrarily as 100 (dashed line). Results are mean ± SD of three independent experiments. Statistical analysis was performed using the Tukey–Kramer multiple comparisons test. * *p* < 0.05, *** *p* < 0.001, vs. untreated cells.

Then, the viability of Nthy-ori-3-1 cells after prolonged exposure (9, 12 and 18 days) to MeHg was investigated. A significant increase in the cell viability using 0.1 µM of this metal was noted, with the strongest effect detected after 18 days with an increase of cell proliferation of about 100% respective to untreated cells (Figure 4).

## 3. Discussion

Trace amounts of metals are essential nutrients, but, at high concentrations, they can be toxic for living cells, behave as endocrine disruptors, perturb the hormonal system, and, sometimes, act as carcinogens, mainly depending on dosage and time of exposure [10,11]. Heavy metals are naturally present in the environment in various vehicles at different concentrations in different areas. Higher traces of heavy metals are detectable in proximity to emission sources, including natural geogenic emission and pollution secondary to human activities, from which they reach human tissues by food, water and atmosphere [10]. Recently, the exposure to specific environmental contaminants, such as dioxins, phthalates, pesticides and heavy metals, has been considered an additional causative factor to explain the increased incidence of thyroid cancer [11,12]. In particular, an increased risk of thyroid malignancies has been observed in some volcanic areas, such as Hawaii, New Caledonia, French Polynesia, Iceland and Sicily (Italy) [13,14], and high levels of Hg, as well as other toxic metals, have been detected in the drinking water of a volcanic areas in Sicily [7]. Even in another volcanic area with a high incidence of thyroid cancer, French Polynesia, high levels of daily Hg intake in the population were found, coming mainly from the fisheries [8]. However, a carcinogenic effect of specific heavy metals has never been tested directly on thyroid cells.

In this work, we analyzed the effects of MeHg on the human non-tumorigenic thyroid cells, Nthy-ori-3-1, and found a growth promoting action when MeHg was used for 24 h at the concentration of 0.1 µM, whereas exposure of the cells to higher concentrations determined a significant decrease of cell viability. Moreover, the promotion of cell proliferation was more evident when the cells were treated with low doses of MeHg for a prolonged time. Indeed, it is known that the action of mercury compounds as major pollutants is strictly related to its tendency to accumulate in tissues and organs of animals [15]. A growth promoting action of MeHg at low dosage was reported in other experimental models in vitro [16], but not in thyroid cells. To our knowledge, there are no studies which have investigated the molecular mechanism on the basis of the effects of mercury on human thyroid cell proliferation and/or potential genetic or epigenetic alterations which may explain the promotion of benign, as well as malignant, tumors: in fact, the oxidative stress and the ability to alter cellular metabolic pathways can favor the transformation of susceptible cells [17]. However, in our experimental conditions, no evidence of effects on ROS production was observed at low doses of MeHg. Conversely, an increase in the levels of phosphor-ERK was observed, suggesting the involvement of the MAPK-mediated pathway in the promotion of cell growth. It must be noted that dysregulation of this pathway is implicated in the transforming action of the most frequent genetic alterations occurring in thyroid neoplasia [18,19]. Thus, although our findings do not demonstrate a direct tumorigenic effect on thyroid cells, they suggest, as a mechanistic explanation of the growth dysregulation of low sub-toxic concentrations of MeHg, the activation of the ERK signaling pathway. Further studies will help to identify the molecular target of MeHg action among the various elements of ras-RAF-MEK-ERK cascade. Moreover, the present results obtained after a longer exposure, support the hypothesis of a carcinogenic activity of Hg exerted by the bioaccumulation occurring after a prolonged exposure to of this contaminant, when its presence in the environment is over the safety levels. In addition, the possibility of a synergistic action of even low concentrations of mixtures of heavy metals must also be considered [10]. Further studies, also using in vivo experimental models, will be necessary to better characterize the action of this contaminant on thyroid cells.

## 4. Materials and Methods

### 4.1. Cell Culture and Cell Proliferation Assay

Human non-tumorigenic thyroid Nthy-ori-3-1 cells were cultured in RPMI 1640 (Thermo Fisher Scientific Inc., Waltham, MA, USA) and maintained at 37 °C in a humidified 5% CO_2_ incubator, as previously described. Briefly, cells were seeded in 96 well plates at a density of 6 × 10^3^ and after 24 h, growth medium was replaced by fresh normal medium supplemented with MeHg (concentration range from 0.1 to 5 µM). After a short (24 h) and a prolonged exposure (9, 12, 18 days), cell proliferation was assessed by MTT assay [20]. A microplate spectrophotometer (xMark, Biorad, Milan, Italy) at a wavelength of 540 nm and a reference wavelength of 690 nm were used to quantify the solubilized product. Results are expressed as percentages over untreated cells (control).

### 4.2. Cell Cycle Assay

Cell cycle distribution was analyzed by flow cytometry as follows [21]. Nthy-ori-3-1 cells (100 × 10^3^) were seeded in 6-well plates and the next day were treated with MeHg (0.1, 0.5, 1, 2.5 and 5 µM). After 24 h of incubation, cells were harvested, washed with PBS and fixed in 70% cold ethanol at −20 °C overnight. Then, cells were washed and incubated with PBS containing 0.1% Triton X-100, 20 µg/mL propidium iodide and 0.05 µg/mL RNase A (Merck Life Science S.r.l., Milan, Italy) for 30 min at 37 °C. The Accuri™ C6 Plus personal flow cytometer was used to analyze the stained cells (Becton Dickinson, San Jose, CA, USA).

### 4.3. ROS Assay

Nthy-ori-3-1 cells were treated for 0.5, 1, 3 and 6 h with MeHg 0.1, 0.5, 1, 2.5 and 5 µM. At the end of the treatment, the cells were resuspended in phenol red-free medium containing a fluorescent probe H2DCF-DA (25 mM) and cultured for 30 min at 37 °C. Then, cells were incubated for an additional 30 min in the presence or not of H_2_O_2_ (100 µM) [22]. Fluorescence was evaluated by flow cytometric analysis using the Accuri™ C6 Plus personal flow cytometer (Becton Dickinson, San Jose, CA, USA).

### 4.4. Western Blot Analysis

The extraction of total proteins was performed as previously described [23]. After 6 h of exposure to low doses of MeHg (0.1, 0.5, 1 µM), 20 µg of proteins were run on a 12% SDS-polyacrylamide gel, transferred to Hybond-PVDF membranes (VWR, Milan, Italy), blocked with TTBS/milk (TBS, 1% Tween 20 and 5% non-fat dry milk) and incubated overnight with each of the following antibodies: polyclonal anti-Akt (cod. 9272) and anti-phospho-Akt (4058) antibodies (Cell Signaling, Euroclone, Milan, Italy) diluted to 1:1000 and 1:500 respectively, polyclonal anti-ERK antibody (sc-154) diluted 1:1000, monoclonal anti-phospho-ERK antibody (sc-7383) diluted 1:500 (Santa Cruz Biotechnology, DBA, Segrate, Milan, Italy) and monoclonal anti-GAPDH antibody (AM4300) diluted 1:40.000 (Thermo Fisher Scientific). The membranes were incubated with horseradish goat anti-mouse or goat anti-rabbit HRP-conjugated antibodies from Transduction Laboratories (Lexington KY, USA) in TTBS/milk. The blots were developed with ECL-PRIME reagent (Perkin Elmer, Monza, Italy).

### 4.5. RNA Extraction and Real-Time PCR

The Trizol method (Thermo Fisher Scientific Inc) was used to extract total RNA from cells treated or not with MeHg. *NIS*, *TG, TPO* and *TSHR* genes levels were determined with a real-time quantitative reverse transcriptase-polymerase chain reaction (RT-PCR), as previously described [24]. Then, 1 μg of total RNA was reverse-transcribed with a High-Capacity cDNA Reverse Transcription Kit (Thermo Fisher Scientific). The cDNAs were amplified in an Applied Biosystems 7900HT fast real-time PCR Sequence Detection System (Thermo Fisher Scientific Inc) using fast quantitative PCR thermal cycler parameters. A 20 µL reaction contained 20 ng of cDNA, 10 μL TaqMan Fast Universal PCR master mix (Thermo Fisher Scientific) and 2 μL of a primer/probe mixture for each gene evaluated. The *β-actin* gene was used as an endogenous reference. All amplification reactions were performed in triplicate, and the threshold cycles (identified with Thermo Fisher Scientific software, SDS 2.4 software) of the 3 reactions were averaged. The detectability threshold was set to 37. Results were obtained by the 2^−ΔΔ*C*t^ method and normalized to a sample of control cells or normal tissues.

### 4.6. Statistical Analysis

GraphPad Prism version 5.0 statistical software (GraphPad Software Inc., San Diego, CA, USA) was used. A one-way ANOVA followed by the Tukey–Kramer multiple comparisons test were adopted for statistical analysis. The results are expressed as means ± standard deviation (SD).

## 5. Conclusions

Finally, we have demonstrated that MeHg, at low doses and especially after a prolonged exposure, as may occur after prolonged exposure to this environmental contaminant, exerts a promoting effect on thyroid cell proliferation, by acting on the ERK-mediated pro-oncogenic signal transduction pathway.

## Supplementary Materials

Supplementary materials can be found at https://www.mdpi.com/1422-0067/21/5/1556/s1.
