# Supplementary material for: Low Doses of Methylmercury Induce the Proliferation of Thyroid Cells In Vitro Through Modulation of ERK Pathway
Source: Int J Mol Sci. 2020 Feb 25;21(5):1556. doi: 10.3390/ijms21051556 (PMC7084424; doi:10.3390/ijms21051556)
Supplement: Supplementary file 1 [file ijms-21-01556-s001.pdf]

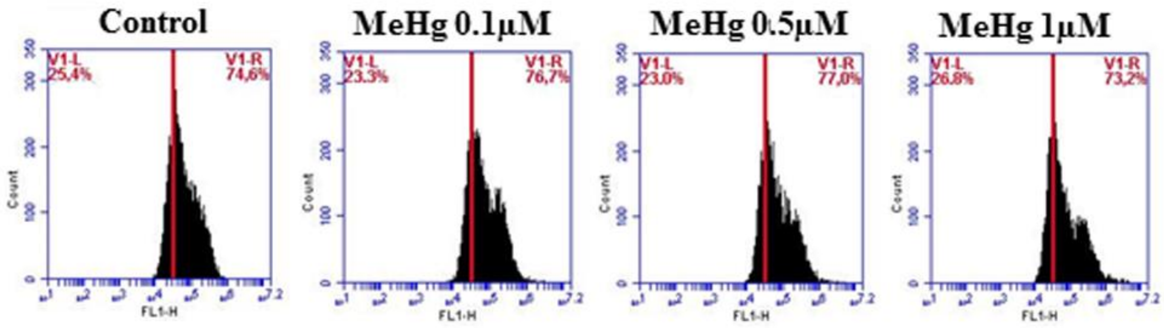

Figure S1. Determination of ROS production by cytofluorimetric analysis in Nthy-ori-3-1 cells. Cells were pretreated with MeHg (0.1, 0.5 and 1 μM), then incubated with H_2DCF-D and the fluorescence of DCF was evaluated by flow cytometry. Hydrogen peroxide (250 μM) induced accumulation of intracellular ROS. Results of a representative experiment of three separate determinations are shown.

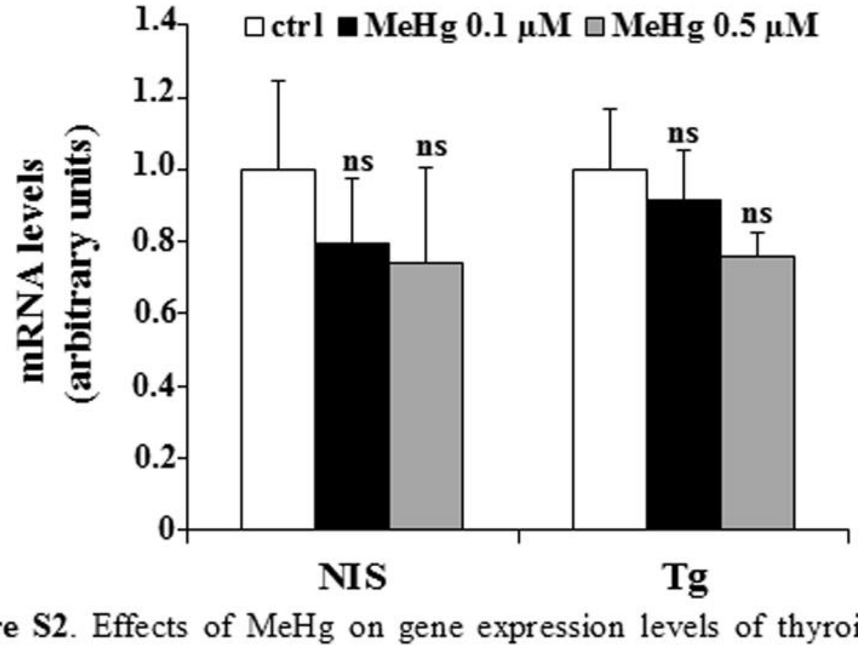

Figure S2. Effects of MeHg on gene expression levels of thyroid differentiation markers. *NIS* and *thyroglobulin* (*Tg*) mRNA levels in Nthy-ori-3-1 cells after treatment with MeHg (0.1 and 0.5 μM). Values are expressed as the mean ± SD. Statistical analysis was performed using the Tukey-Kramer multiple comparisons test. p values upper than 0.05 were considered not statistically significant (ns).
